# The effect of *Psoroptes ovis* infestation on ovine epidermal barrier function

**DOI:** 10.1186/1297-9716-44-11

**Published:** 2013-02-11

**Authors:** Miriam R Stoeckli, Tom N McNeilly, David Frew, Edward J Marr, Alasdair J Nisbet, Adri HM van den Broek, Stewart TG Burgess

**Affiliations:** 1 Moredun Research Institute, Pentlands Science Park, Bush Loan, Edinburgh, Midlothian, Scotland, EH26 0PZ, United Kingdom; 2 The Royal (Dick) School of Veterinary Studies, The University of Edinburgh, Midlothian, Scotland, EH25 9RG, United Kingdom; 3 The Roslin Institute, The University of Edinburgh, Division of Veterinary Clinical Sciences, Hospital for Small Animals, Easter Bush Veterinary Centre, Roslin, Midlothian, Scotland, EH25 9RG, United Kingdom

## Abstract

Sheep scab is an intensively pruritic, exudative and allergic dermatitis of sheep caused by the ectoparasitic mite *Psoroptes ovis*. The purpose of the present study was to investigate the effect of *P. ovis* infestation on different components of the ovine epidermal barrier within the first 24 hours post-infestation (hpi). To achieve this, the expression of epidermal differentiation complex (EDC) genes and epidermal barrier proteins, the nature and severity of epidermal pathology and transepidermal water loss (TEWL) were evaluated.

By 1 hpi a significant dermal polymorphonuclear infiltrate and a significant increase in TEWL with maximal mean TEWL (598.67 g/m^2^h) were observed. Epidermal pathology involving intra-epidermal pustulation, loss of epidermal architecture and damage to the basement membrane was seen by 3 hpi. Filaggrin and loricrin protein levels in the stratum corneum declined significantly in the first 24 hpi and qPCR validation confirmed the decrease in expression of the key EDC genes *involucrin*, *filaggrin* and *loricrin* observed by microarray analysis, with 5.8-fold, 4.5-fold and 80-fold decreases, respectively by 24 hpi.

The present study has demonstrated that early *P. ovis* infestation disrupts the ovine epidermal barrier causing significant alterations in the expression of critical barrier components, epidermal pathology, and TEWL. Many of these features have also been documented in human and canine atopic dermatitis suggesting that sheep scab may provide a model for the elucidation of events occurring in the early phases of atopic sensitisation.

## Introduction

Sheep scab is an intensely pruritic, exudative, allergic dermatitis caused by the ectoparasitic mite *Psoroptes ovis*. It is a highly contagious disease and of major economic and welfare importance to the UK sheep industry [1-4]. The surface-living mite deposits excretory and secretory products containing homologues of known house dust mite (HDM) allergens and enzymes onto the stratum corneum [5,6]. Clinical observation and histological studies have shown that by 24 hours post-infestation (hpi) of naïve sheep these allergens and enzymes have penetrated the stratum corneum and initiated an intense immunoinflammatory response [7] that culminates in immediate IgE-mediated [8,9] and delayed, cell-mediated, hypersensitivity reactions [9]. This early, intense inflammatory response appears to be critical in determining the direction of the subsequent acquired immune response and is dependent on the rapid penetration of mite products through the epidermal barrier.

Integrity of the skin barrier is critical in preventing excessive transepidermal water loss (TEWL) and in skin defence by reducing penetration of exogenous molecules including allergens [10]. The stratum corneum plays a pivotal role in skin barrier function and is composed of anucleated corneocytes, embedded in an extracellular lipid matrix [11] and encased by a cornified envelope consisting of cross-linking proteins such as filaggrin, loricrin and involucrin and free amino acids, a combination forming what has been termed natural moisturising factor (NMF) [12,13]. Among the allergens and enzymes deposited on the stratum corneum by *P. ovis* are *Pso o 1* and *Pso o 3* which are, respectively, homologues of the proteolytic enzymes *Der p 1* (cysteine protease) and *Der p 3* (serine protease) produced by the HDM *Dermatophagoides pteronyssinus* and Sar s 3 (serine protease) produced by the scabies mite *Sarcoptes scabiei*[14]. By analogy, proteolytic activity of these enzymes may disrupt the epidermal barrier by cleavage of tight junctions [15,16] and digestion of filaggrin [14] increasing barrier permeability and TEWL, leading to the initiation of a skin drying cycle and enabling the ingress of exogenous allergens [17].

In addition, recent transcriptome microarray analysis of the ovine cutaneous response up to 24 hpi with *P. ovis* demonstrated significant alterations in the expression of genes located in the epidermal differentiation complex (EDC) including significant down regulation of *filaggrin*, *loricrin* and *involucrin*[18]. Earlier microarray analyses revealed similar alterations in the expression of EDC genes in human atopic dermatitis, and notably the repressed expression of *filaggrin*, *loricrin* and *involucrin*[19,20]. The importance of these proteins in barrier function is indicated by the observation that loss of function mutations in *filaggrin*, the most intensively studied EDC gene, are associated with diseases with compromised epithelial barrier function such as atopic dermatitis, ichthyosis, psoriasis and asthma [21]. Skin barrier function has been traditionally assessed by measurement of TEWL [22,23] which is increased in non-lesional and lesional skin of atopic humans [24,25] and dogs [26] compared to healthy individuals.

It is clear that *P. ovis*-derived enzymes and allergens are able to penetrate the skin barrier initiating a dynamic, multi-faceted immunoinflammatory response involving alterations in the expression of a wide range of genes and proteins including those of the EDC [18]. As early events appear to be of critical importance in the establishment of a sheep scab infestation, the purpose of the present study was to focus on the first 24 hpi, investigating the effect of mite products on different components of the ovine epidermal barrier – a study not reported previously. Here we demonstrate early and significant changes in the expression of selected EDC genes following exposure of sheep skin to *P. ovis*. We employed immunohistochemistry to evaluate the expression of filaggrin and loricrin at the protein level in the stratum corneum and document the nature and severity of histological changes in the skin following infestation. Finally we assessed the influence of these mite-induced changes on skin barrier function through the measurement of TEWL. An understanding of these initial events may provide new insights into the pathogenesis of sheep scab and of other allergic diseases such as atopic dermatitis that compromise epidermal barrier function and integrity.

## Materials and methods

### Animal study, *P. ovis* infestation and skin biopsy collection and fixation

Ethical approval for this study was obtained from the Moredun Research Institute (MRI) Experiments Committee and animals were monitored daily in accordance with guidelines agreed with the UK Home Office. *P. ovis* mites (mixed population consisting of adults, nymphs and larvae) were harvested from infested donor animals maintained at MRI. Sheep scab naïve, ~9 month old Scotch mule lambs (*n* = 9) were maintained at MRI. Immediately prior to infestation with *P. ovis*, an area of the left flank (50 cm (long) × 20 cm (deep)) of each animal was shaved to the skin with electric clippers and two plastic isolation chambers (1 cm deep × 1 cm diameter) were adhered to the skin with surgical glue (Indermil, Henkel, Dusseldorf, Germany). After one hour to allow glue drying and chamber adhesion and following the administration of a local anaesthetic (1 mL, 2% (w/v) lignocaine hydrochloride and 0.001% (w/v) adrenaline (Lignol, Arnolds, Harlescott, UK)) two skin biopsies were removed from each animal, one from within each isolation chamber, using a disposable 8 mm biopsy punch (Henry Schein Animal Health, Dumfries, UK), these samples represented time 0 (uninfested). Following biopsy removal the sample site was sealed with Michel surgical suture clips (Henry Schein Animal Health, UK). The skin biopsies were cut into two and one half was fixed in 5 mL, 4% paraformaldehyde in phosphate buffered saline (PBS, pH7.4) for 6 h then transferred and stored in 70% ethanol prior to processing. The other half of each biopsy was placed into 5 mL RNALater solution (Life Technologies Corporation, Paisley, UK) and stored at 4°C overnight prior to storage at −20°C and subsequent RNA extraction. For the time course samples, eight further isolation chambers were adhered to the shaven area of the left flank of each animal as described above. Approximately 20–50 mites were placed directly onto the skin within each chamber and then after carefully removing mites with a cotton bud, biopsies were taken from within two chambers per time point, as detailed above, at the following intervals 1, 3, 6 and 24 hpi. After collection biopsies were cut into two halves and fixed and stored as described above.

### Extraction of RNA from ovine skin biopsies

RNA extraction from thawed skin biopsy samples was performed as described previously [18]. RNA sample quality was assessed on an Agilent Bioanalyser (Agilent Technologies UK, Wokingham, UK) an RNA Integrity Number (RIN) was obtained for each sample and RNA yield was assessed on a ND-1000 Nanodrop spectrophotometer (Thermo Scientific Ltd, Cramlington, UK). RNA samples with a RIN >7.5 were considered to be of acceptable quality for downstream analysis [27]. Duplicate RNA samples from each time point, per animal were then pooled (5 μg RNA from each of 2 replicates for time = 0 and 1, 3, 6 and 24 hpi samples, forming 10 μg total RNA pools per time point/animal).

### Microarray analysis of ovine skin biopsy samples following infestation with *P. ovis*

The experimental details of the microarray study for the analysis of the host response to skin infestation with *P. ovis* mites over a 24 h time course have been described previously [18]. Briefly, this analysis identified 1552 genes significantly differentially expressed (fold-change greater than 1.8 and a false discovery corrected *p*-value < 0.05) at the transcript level over the time course of *P. ovis* infestation [18]. Two clusters of genes implicated in the control of terminal differentiation of keratinocytes were identified and their up- and down-regulation at 24 hpi compared to baseline (0 h) was assessed.

### Quantitative real time PCR (qPCR) validation of selected EDC gene expression

qPCR was used to verify the differential expression of three EDC genes; *filaggrin* (*FLG*), *loricrin* (*LOR*) and *involucrin* (*IVL*) using RNA extracted from the ovine skin biopsies obtained from 6 individual sheep across the 24 h time course of infestation with samples taken at 0 (baseline), 1, 3, 6 and 24 hpi. External primer sets for *FLG* and *IVL* were designed on the bovine gene sequences and used to amplify gene fragments for qPCR standard curve generation, the sequences were as follows: Ext-*FLG*-Forward: 5^′^GAAAGAGGGAAAAAGAGACATGG‘3; Ext-*FLG*-Reverse: 5^′^CCTTCGCTATCGCTGGCCTG’3; Ext-*IVL*-Forward: 5^′^ATACCCAGCAGGAGCAAGTG‘3; Ext-*IVL*-Reverse: 5^′^CTTCTCCTGTTCCAGCTGTCC ‘3. Internal primer sets for *FLG* and *IVL* were also designed on the bovine gene sequences and were as follows: Int-*FLG*-Forward: 5^′^TGGAAGACCTGGTTCAGCTT’3; Int-*FLG*-Reverse: 5^′^TGCTTCCAGATCCAGAGGAG’3; Int-*IVL*-Forward: 5^′^GCCTCAGAAAGCAGAACACC’3; Int-*IVL*-Reverse: 5^′^GACTGGGTGGATCTCTTGGA’3.

Total RNA was used as template for the generation of complementary DNA (cDNA) using Superscript II (Life Technologies Corporation, UK) and anchored oligo (dT) primers (Sigma, UK) according to the manufacturer’s protocols. For the validation of *FLG* and *IVL* a two-step qRT-PCR was performed using SYBR green absolute quantification and the standard curve method. Plasmids and standard curves for the selected genes and the endogenous control glyceraldehyde 3-phosphate dehydrogenase (*GAPDH*) were prepared as previously described [28,29]. Serial 1:10 dilutions of plasmid containing the gene of interest ranging from 10^2^ to 10^8^ copies per μL were run in parallel with each series of samples on an ABI Prism 7000 Sequence Detection System (Life Technologies Corporation, UK) allowing automatic standard curve generation. PCR efficiencies calculated from the slopes were consistently ≥ 95%. The number of copies per μL of sample was calculated and results normalized to the *GAPDH* endogenous control, the expression of which had been shown not to vary significantly with infestation based on the microarray data from the same study [18]. Samples and standards were run in triplicate and melting curve analysis was performed at the end of each PCR to verify product specificity. Validation of *LOR* expression was performed using the Taqman relative quantification, 2-ddCt method [30,31]. As no ovine *LOR* sequence was available at the time of study a pre-validated “assay-on-demand” specific primer and probe set based on bovine *LOR* was used for analysis of skin biopsy RNA (Life Technologies Corporation, UK). cDNA was assayed for *LOR* expression using pre-validated Taqman primers and probeset (Assay ID: Bt03269087_m1, Life Technologies Corporation, UK) and performed in quadruplicate. *LOR* expression results were normalised to *GAPDH* using a Taqman pre-validated “assay-on-demand” primer and probeset based on bovine *GAPDH* and previously shown within our lab to cross react with the ovine gene (Assay ID: Bt03210913_g1, Life Technologies Corporation, UK). Gene expression differences across the 24 h time course of infestation were calculated by relative quantification using time 0 (uninfested skin biopsy RNA samples) as the control compared to the 1, 3, 6 and 24 hpi samples.

### Immunohistochemical labelling and measurement of intensity of fluorescent labelling for filaggrin and loricrin protein in ovine skin sections

Immunohistochemistry was performed on serial 5 μm tissue sections of paraformaldehyde fixed samples. Sections were deparaffinized in xylene and rehydrated in water. Antigen retrieval was performed on sections to be labelled for filaggrin by autoclaving in 10 mM citrate buffer of pH6 at 121°C for 10 min. Subsequently, these sections and those to be labelled for loricrin were rinsed twice in PBS (pH7.4) and washed in 0.5% Tween 80 in PBS (PBST80) for 5 min. Endogenous peroxidase activity was quenched by incubation in 0.3% H_2_O_2_ in PBST80 for 20 min and followed by a 5 min wash in PBST80 before loading into a Sequenza slide rack (Thermo Scientific Ltd, UK). Following a further PBS wash, sections were incubated in 25% normal goat serum (NGS) to block non-specific tissue antigens. Sections were then incubated overnight at 4°C with either, 100 μL mouse monoclonal anti-filaggrin antibody (SPM181, Abcam, Cambridge, UK*)* at 1:25 in 10% NGS/PBST80 or with 100 μL of rabbit polyclonal anti-loricrin antibody (AB85679, Abcam UK) at 1:2000 (0.5 μg/mL) in 10% NGS/PBST80. Overnight incubation of appropriate isotype controls was carried-out in 10% NGS/PBST80 (1:25 dilution mouse isotype control mAb VMP21 or 0.5 μg/mL purified rabbit immunoglobulin (Sigma Aldrich Company Ltd, Gillingham, UK)) for mouse anti-filaggrin and rabbit anti-loricrin primary antibodies respectively. In addition to isotype-controls, controls without antibodies were performed by incubation with 100 μL 10% NGS/PST80. Sections were rinsed twice in PBS prior to incubation with 100 μL of Alexa-fluor 488 goat anti-mouse IgG (1:1000, Life Technologies Corporation, UK) or Alexa-fluor 488 goat anti-rabbit IgG (1:1000, Life Technologies Corporation, UK) in 10% NGS/PBST80 for 1 h in the dark. Slides were mounted in Mowiol® 4–88 (Merck KGaA, Darmstadt, Germany) and stored at 4°C prior to visualisation.

At each time-point (0, 1, 3, and 24 hpi) five random fields of epidermis and superficial dermis were captured at 40× magnification using an AxioCam MRm digital camera mounted on a Axiovert 200 M inverted fluorescence microscope equipped with an ApoTome slider module (Carl Zeiss Ltd, Welwyn Garden City, UK). Images were processed using AxioVision version 4.7.2 digital image processing software (Carl Zeiss Ltd, UK). An identical exposure time was used across all fields/time points. In pictures with patchy fluorescence or loss of fluorescence, composite images were taken using both fluorescence and phase contrast microscopy to identify the epidermis. The mean fluorescence intensity (MFI)/mm epidermis for both filaggrin and loricrin labelling was determined for each tissue section using ImageJ, version 1.42 software [32], with five fields evaluated per section.

### Qualitative and semi-quantitative histopathological analysis

Histopathological analysis was performed on 5 μm sections of paraformaldehyde fixed tissue from all 9 sheep at the following time points (0 h, 1 hpi, 3 hpi and 24 hpi). Sections were stained with haematoxylin and eosin and were mounted in Histomount® (Life Technologies Corporation, UK).

#### Assessment of epidermal changes

On the basis of earlier studies [8,9] a blinded evaluation of the hematoxylin and eosin (H&E) stained sections was undertaken. Three criteria for the histopathological assessment of epidermal change (subcorneal and parafollicular pustulation, disruption of normal structure, loss of basement membrane) were identified. The severity of epidermal changes was classified as mild (< 1/3 of the epidermis affected), moderate (1/3-2/3 of the epidermis affected) and severe (> 2/3 of the epidermis affected).

#### Assessment of dermal infiltrate

The predominant cell type present in the dermal infiltrate was assessed subjectively and classified as either mononuclear or polymorphonuclear. The severity of the infiltrate was categorised as mild, moderate or severe. In addition, polymorphonuclear cells were counted in 25 successive images taken by an Olympus DP72 camera attached to an Olympus BX41 microscope at × 60 magnification. Graticules were entered with imaging software (Cell, Olympus Cell Family, Olympus UK Ltd, Southend-on-sea, UK) to simplify cell counting. Intravascular and intrafollicular polymorphonuclear cells were excluded from the count and large vessels and follicles were subtracted from the total area counted.

### Determination of transepidermal water loss (TEWL)

TEWL was evaluated with a closed chamber VapoMeter (Delfin Technologies Ltd, Kuopio, Finland) using the standard instrument adaptor (diameter = 11 mm) with readings recorded in g/m^2^h. For this study four additional sheep were infested with *P. ovis* (20–50 mites) on two adjacent sites on the left flank. Prior to infestation, three measurements of TEWL were taken on a shaven area of the flank of each animal (representing the time 0 or baseline sample). Following infestation a further three measurements of TEWL were taken at the two sites of infestation on each sheep at 1, 3, 6 and 24 hpi. Average values for each animal at each time point were obtained by calculating the mean of the repeated measurements across both infestation sites. In addition, further measurements were taken from uninfested skin adjacent to the infestation sites at each of the four time points to ensure accuracy of measurements.

### Statistical analysis

All statistical analyses were performed using GraphPad Prism software (Version 5, GraphPad Software Inc, La Jolla, USA). qPCR, fluorescent intensity, PMN cell count and TEWL data were found to be normally distributed and data analyses were performed using a repeated measures one-way ANOVA analysis with a Tukey post-hoc test. *P*-values of < 0.05 were considered significant.

## Results

### Microarray analysis of ovine skin following *P. ovis* infestation

**Figure 1:**
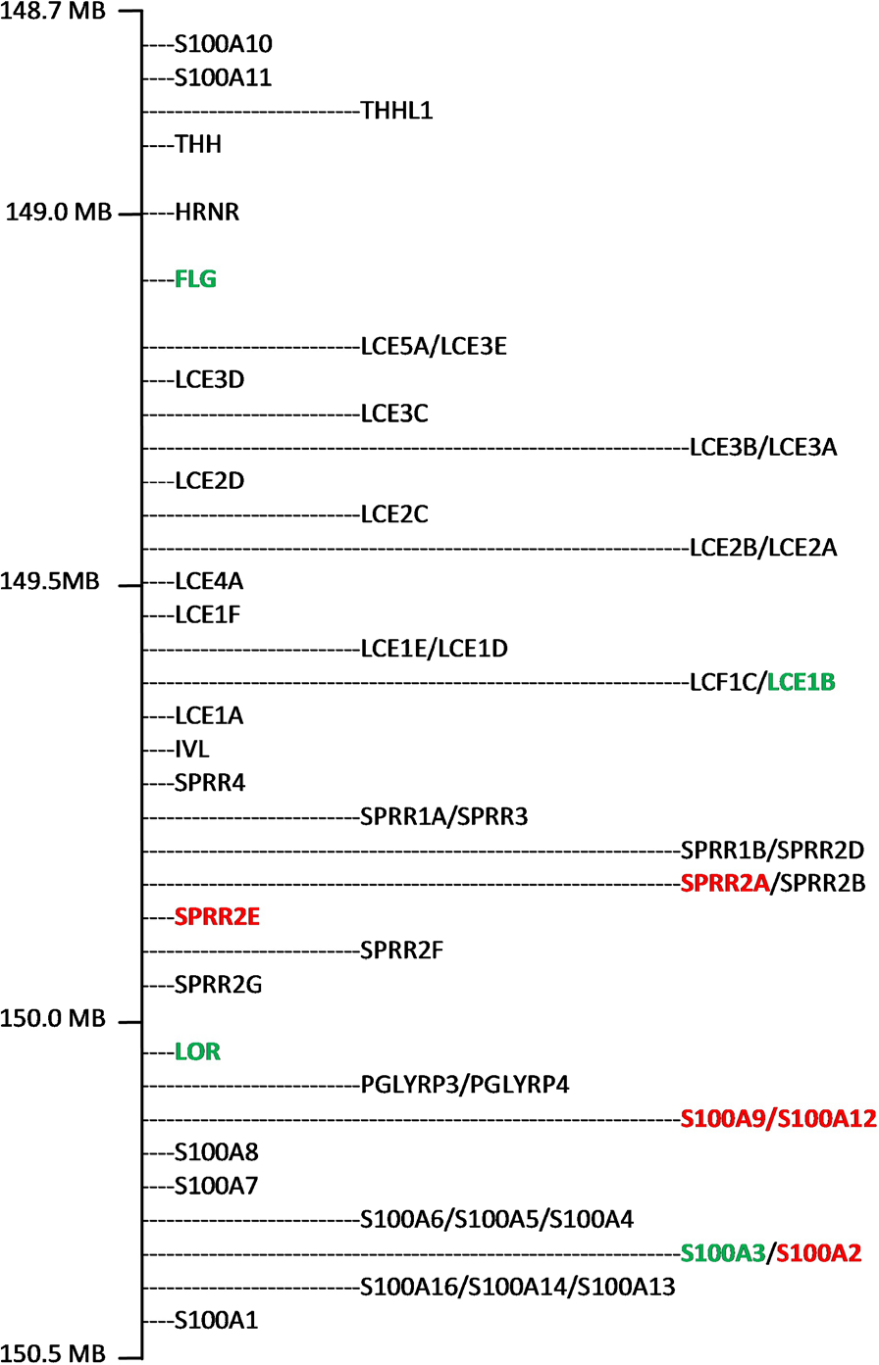
**EDC gene cluster (based on the human genome) located within chromosome 1q21.** Microarray analysis of the transcriptomic response in sheep skin exposed to *P. ovis* revealed that the following genes were differentially expressed by 24 hpi compared to baseline samples: FLG = 3-fold down; *LOR* = 26-fold down; *LCE1B* = 2-fold down; *S100A3* = 2-fold down; *S100A9* = 90-fold up; *S100A12* = 60-fold up; *S100A2* = 3-fold up; *SPRR2E* = 160-fold up; *SPRR2A* = 320-fold up. Red = up-regulated, green = down-regulated over the time course of infestation.

Two clusters of EDC genes, implicated in the control of terminal differentiation of keratinocytes, were significantly differentially expressed in ovine skin across the 24 h time course of infestation with *P. ovis* (Figure 1). The first cluster contained EDC genes with increased expression across the time course of infestation with peak expression by 24 hpi and included the small proline rich proteins (SPRRs) *SPRR2A* (324-fold increase) and *SPRR2E* (average 136-fold increase) and the S100 calcium binding proteins, *S100A9* (90-fold increase) and *S100A12* (66-fold increase). The second cluster contained EDC genes with decreased expression by 24 hpi and included *LOR* (26-fold decrease), *FLG* (5.8-fold decrease), S100 calcium binding protein A3 (*S100A3*, 2-fold decrease) and late cornified envelope 1B (*LCE1B*, 2-fold decrease).

### qPCR validation of selected EDC gene expression

**Figure 2:**
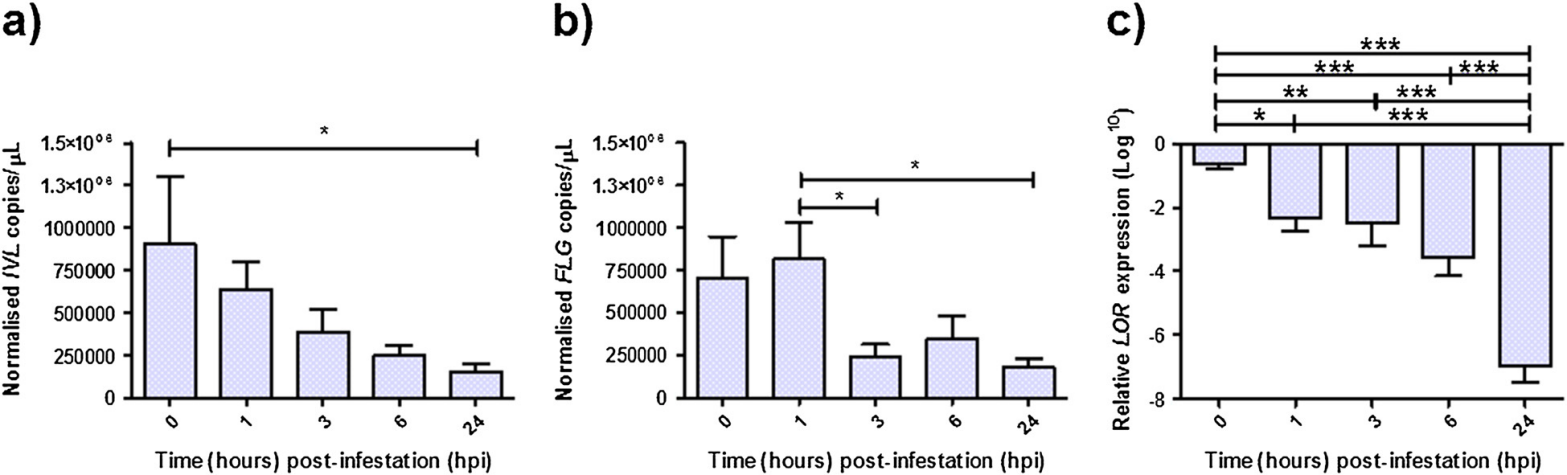
**qPCR validation of *IVL*, *FLG *and *LOR *expression in ovine skin following *P. ovis *infestation.** Expression data across six individual sheep for *IVL* (**a**) *FLG* (**b**) and *LOR* (**c**). Values for *IVL* and *FLG* were normalised to the expression of the ovine housekeeping gene *GAPDH* and expressed as copies/μL at each time point across six animals. *LOR* gene expression was assessed using the relative quantification ddCt method and normalised to the time = 0 sample. * = *p* < 0.05, ** = *p* < 0.01, *** = *p* < 0.001. Error bars show standard error of the mean (SEM).

*IVL* (Figure 2a) was significantly differentially expressed by 24 hpi with a 5.8 fold decrease compared to time 0 (*p ≤* 0.05). *FLG* (Figure 2b) was also significantly differentially expressed by 3 (*p ≤* 0.05) and 24 hpi (*p ≤* 0.05) with respectively, a 3.48-fold and 4.46-fold decrease compared to 1 hpi. *LOR* gene expression (Figure 2c) was the most significantly down-regulated of the three EDC genes assessed with an 80-fold reduction in expression at 24 hpi compared to time 0 (*p ≤* 0.001).

### Immunohistochemical labelling of filaggrin and loricrin protein in ovine skin sections

**Figure 3:**
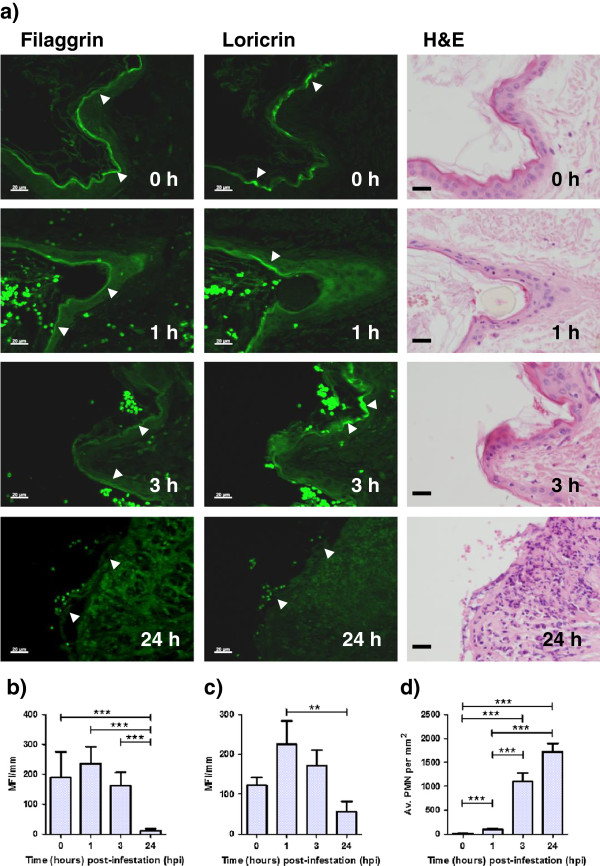
**Immunohistochemical labelling of filaggrin and loricrin and changes in skin histopathology following *P. ovis *infestation.** (**a**) Representative images of fluorescence immunohistochemical (IHC) labelling of filaggrin and loricrin and H&E stained sections from one sheep at time 0, 1, 3 and 24 hpi. Scale bars represent 20 μm. Mean fluorescence intensity (MFI)/mm epithelium for filaggrin (**b**) and loricrin (**c**) IHC labelling at time 0, 1, 3 and 24 hpi. (**d**) Mean polymorphonuclear cell counts/mm^2^ at time 0, 1, 3 and 24 hpi. ** = *p* < 0.01, *** = *p* < 0.001. Error bars show standard error of the mean (SEM).

Representative images of immunohistochemical labelling of filaggrin and loricrin are shown in Figure 3a, with the MFI/mm epidermis for filaggrin and loricrin labelling over the time-course of infection shown in Figures 3b and c, respectively. One of the nine sheep used had to be excluded as significant background staining affected the measurements. The ranges and averages of the mean fluorescence intensity/mm (MFI/mm) of the remaining eight sheep for filaggrin and loricrin at 0 h, 1 hpi, 3 hpi and 24 hpi are shown in Table 1. There were significant differences in filaggrin staining between time 0 and 24 hpi (*p* < 0.001), between 1 and 24 hpi (*p* < 0.001) and between 3 and 24 hpi (*p* < 0.001) (Figure 3b). Fluorescent intensity for loricrin at 24 hpi was significantly lower (*p* < 0.01) than at 1 hpi (Figure 3c).

**Table 1:** Immunohistochemical labelling (filaggrin, loricrin), histopathology and transepidermal water loss

**Time**	**0 h**	**1 hpi**	**3 hpi**	**6 hpi**	**24 hpi**
Filaggrin (average MFI/mm)	189 (34.43-751.01)*	236.16 (93.57-469.30)*	160.95 (23.69-370.62)*	N/A	11.49 (0–44.59)*
Loricrin (average MFI/mm)	122.06 (83.79-227.59)*	225.15 (69.60-402.44)*	171.71 (0.09-305.21)*	N/A	55.28 (0–213.95)*
Mean PMN cell count per mm^2^	9.13 (0.97-31.91)*	92.09 (12.57-207.93)*	1099.83 (410.06-1959.38)*	N/A	1174.16 (1098.65-2330.75)*
TEWL (g/m^2^h)	20.54 (13.62-29.48)*	598.67 (598–662)*	585.17 (597.67-637)*	516.46 (403.83-597.33)*	396.75 (331.17-481)*

Mean fluorescence intensity (MFI)/mm epithelium for filaggrin and loricrin and mean polymorphonuclear (PMN) cell counts /mm2 at time 0, 1, 3 and 24 hours post-infestation (hpi). Transepidermal water loss (TEWL) g/m^2^h at 0,1,3,6 and 24 hpi. *Reference range.

### Quantitative and semi-quantitative histopathological analysis

#### Epidermal changes

Details of epidermal changes and their severity at 3 hpi and 24 hpi are summarized in Table 2, and representative images of H&E sections from one animal over the time-course of infection are shown in Figure 3a. Epidermal changes were first observed at 3 hpi with 3/9 biopsies showing mild to moderate pustule formation, 7/9 biopsies showing mild to moderate loss of epidermal architecture and 7/9 biopsies with severe disruption of the basement membrane. By 24 hpi epidermal changes were generally more severe, with 7/9 biopsies exhibiting severe pustule formation or loss of epidermal architecture and 3/9 biopsies exhibiting severe loss of the epidermal basement membrane. No evidence of epidermal hyperplasia was detected at 3 or 24 hpi.

**Table 2:** Epidermal changes and their severity at 3 and 24 hpi

**Time**	**Epidermal pathology**	**Severity**		
		**Mild**	**Moderate**	**Severe**
3 hpi	Pustule formation	1 case	2 cases	0 cases
	Loss of architecture	6 cases	1 case	0 cases
	Loss of basement membrane	1 case	1 case	7 cases
24 hpi	Pustule formation	0 cases	2 cases	7 cases
	Loss of architecture	1 case	1 case	7 cases
	Loss of basement membrane	1 case	3 cases	3 cases

Results of epidermal changes of 9 sheep at 3 and 24 hours post-infestation (hpi) with *P. ovis*. The severity of epidermal changes was classified as mild (< 1/3 of the epidermis affected), moderate (1/3-2/3 of the epidermis affected) and severe (> 2/3 of the epidermis affected).

#### Dermal infiltrate of polymorphonuclear cells

Mean polymorphonuclear (PMN) cell counts per mm^2^ at time 0 (baseline), 1, 3 and 24 hpi are presented in Table 1 and Figure 3d. All polymorphonuclear cells were counted and comprised predominantly eosinophils. There was a significant increase in polymorphonuclear cells in × 60 microscopic fields between time 0 and 1 hpi (*p* < 0.001), time 0 and 3 hpi (*p* < 0.001) and time 0 and 24 hpi (*p* < 0.001). Furthermore, there was a significant increase between 1 and 3 hpi (*p* < 0.01) and between 1 and 24 hpi (*p* < 0.01).

### Trans-epidermal water loss (TEWL)

**Figure 4:**
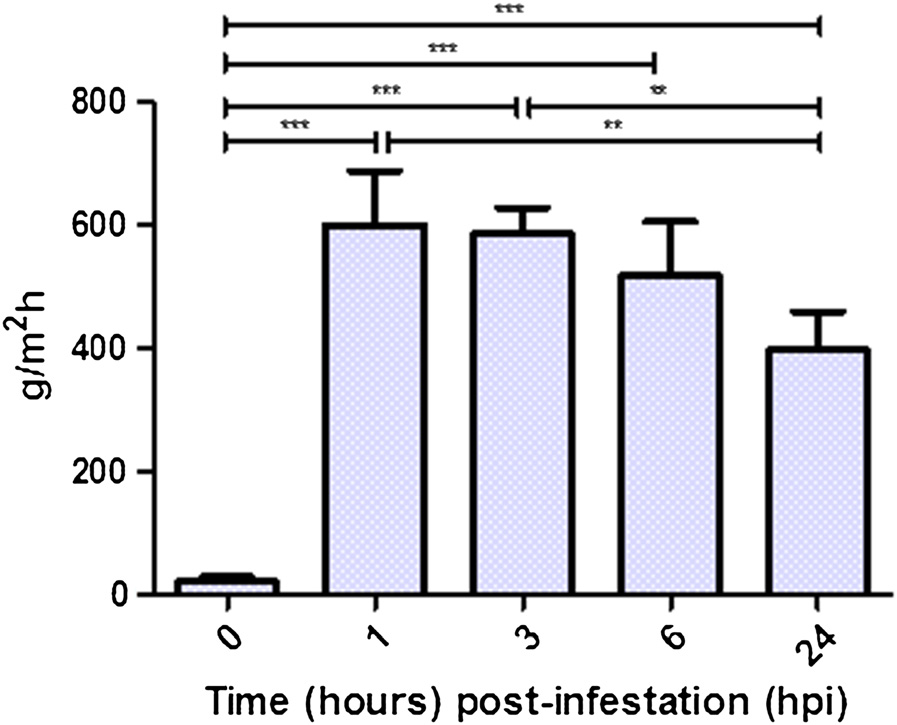
**Trans-epidermal water loss (TEWL) following infestation of sheep skin with *P. ovis*.** TEWL expressed as grams per square meter per hour, values represent the mean values across four sheep, over two independent sites with triplicate measurements from each site at each time point. *** = *p* < 0.005, ** = *p* < 0.01. Error bars show standard deviation of values across all samples (*n* = 4).

Mean TEWL (g/m^2^h) at time 0 (baseline), at 1, 3, 6 and 24 hpi are presented in Table 1 and Figure 4. There were significant changes between time 0 and 1 hpi (*p* < 0.001) time 0 and 3 hpi (*p* < 0.001) time 0 and 6 hpi (*p* < 0.01) and time 0 and 24 hpi (*p* < 0.01). There were also significant differences between 1 and 24 hpi (*p* < 0.01) and 3 and 24 hpi (*p* < 0.01).

## Discussion

In this study we have demonstrated that within 24 h of infestation, *P. ovis* triggers a significant decrease in expression of selected EDC genes and initiates significant breakdown of the epidermal barrier function evidenced by reduced levels of selected EDC proteins at the epidermal surface, cutaneous histopathology and significant TEWL.

As reported earlier, transcriptome microarray analysis of the ovine cutaneous response to *P. ovis* demonstrated that expression of a cluster of EDC genes including those for *FLG*, *LOR* and *IVL* was decreased with maximal repression by 24 hpi [18]. qPCR, employed in the present study, confirmed the decreases in *IVL*, *FLG*, and *LOR* gene expression with 5.8-fold, 4.46-fold and 80–fold decreases, respectively by 24 hpi compared to time 0 (baseline) samples in ovine skin. The canonical Th2 cytokines, IL4 and IL13 have been shown to be over expressed in atopic skin [20,33,34] and are able to trigger down-regulation of *FLG*[35,36], *LOR*[20], *IVL*[20] in human keratinocytes. There is also a growing body of evidence to support a role for the Th2-associated transcription factor STAT6 in this pathway [37]. Therefore, atopic patients may acquire a filaggrin deficiency and the resulting epidermal barrier dysfunction as a result of a Th2-driven inflammatory response. A similar bias towards a Th2 dominated immune response has been demonstrated by transcriptomic analysis of the ovine response to *P. ovis* infestation [18] and suggests that Th2-cytokines may also be involved in suppression of filaggrin synthesis in *P. ovis* infested skin.

In contrast to acquired filaggrin deficiency, reduced *FLG* expression can also be inherited by loss-of-function mutations in *FLG*, which have been demonstrated in patients with atopic dermatitis, psoriasis and ichthyosis vulgaris [38] and potentially occur in canine atopy [39]. However, mutations in *FLG* have not yet been demonstrated in sheep but breed variation in susceptibility to sheep scab has been reported and although only anecdotal may conceivably be related to EDC gene mutations/polymorphisms [40].

The present study recorded a significant decline in the fluorescent intensity of staining for both filaggrin and loricrin protein in the stratum corneum by 24 hpi demonstrating the involvement of these integral constituents of the cornified envelope in the pathology of sheep scab. This reduction in staining intensity may be attributable to active down-regulation of gene expression, potentially as a consequence of the Th2-dominated immune response as previously discussed, and the consequent reduction in protein synthesis. However, as the decrease in both filaggrin and loricrin gene expression and protein levels mirrored the development of pathological changes within the epidermis, it is also possible that loss of keratinocytes, the principal cellular source of these proteins [12], contributed to the reduced levels of filaggrin and loricrin observed in this study. Nevertheless, it is unlikely that keratinocyte loss is solely responsible as increased expression of the keratinocyte-specific EDC genes, *SPRR2A* and *SPRR2E*, was observed at 24 hpi, suggesting that significant numbers of viable keratinocytes were present even when levels of filaggrin and loricrin were lowest. Another possible mechanism for the reduced levels of filaggrin protein is active cleavage of protein by mite proteases: earlier in vitro studies have shown that a recombinant scabies mite protease, *Sar s 3*, a homologue of *Der p 3* and *Pso o 3*, is capable of cleaving filaggrin and that filaggrin protein can be immunolocalised to the scabies mite digestive tract [14]. Therefore, the rapid reduction in fluorescent intensity observed in the present study may have resulted from a combination of reduced protein synthesis due to active down-regulation of gene expression, loss of keratinocytes as a result of *P. ovis* induced pathology and the action of mite-derived proteolytic enzymes.

Histopathological studies have demonstrated that ticks provoke an influx of inflammatory cells within one to 24 h of attachment to tick-naïve hosts [41-43]. Although, in contrast to ticks, there is no evidence that the mouthparts of *P. ovis* penetrate the epidermis [44,45] the present study has established that by 1 hpi, *P. ovis* provokes a significant dermal infiltrate of polymorphonuclear cells dominated by eosinophils and after three hours intra-epidermal pustule formation was detectable along with a loss of epidermal architecture and damage to the basement membrane. These observations are consistent with an earlier report of *P. ovis* causing a significant dermal infiltrate, comprising principally eosinophils, accompanied by and neutrophils, 24 h after infestation of naïve sheep [46]. Although a consistent feature 4 days post infestation with *P. ovis*[46], epidermal hyperplasia was not observed in the present study probably due to the very early nature (1–24 hpi) of the lesions examined [47]. In calves the histological responses to *P. ovis* infestation, although similar to those described in sheep, appear to develop more slowly with only minimal histological changes reported 7 days after the application of two doses of mites at an interval of 21 days [48].

The rapid infiltration of polymorphonuclear cells, observed in the present investigation, is consistent with the presence of potent chemoattractants in *P. ovis* products. The HDM, *D. pteronyssinus*, like *P.* ovis, is a member of the Order Astigmata and in vitro studies have demonstrated the eosinophilic and neutrophilic properties of enzymes, notably *Der p 1, 3* and *9*, produced by these mites and the ability of a HDM extract to induce expression of interleukin-8 (*IL8)* and tumour necrosis factor alpha (*TNF*), both of which are potent neutrophil chemoattractants produced by epithelial cells [49-51]. In vitro studies have reported the rapid induction of ovine keratinocyte *IL8* expression by *P. ovis* mite washes and whole mite extract and the eosinophil chemoattractant properties of *P. ovis* whole mite extract, and *P. ovis* homologues of HDM chemoattractants, such as, *Pso o 1, 3* and *9* have been identified [18,52,53]. These mite products are deposited on the epidermis, probably in the form of faecal pellets, and by analogy with HDM products are likely responsible for the rapid and pronounced infiltration of polymorphonuclear cells documented in the present study. The other aspects of epidermal pathology observed may be caused by the considerable array of enzymes and biologically active compounds present in mite products and also during the host inflammatory response to infestation [54].

The present study also documented the rapid effect of *P. ovis* infestation on epidermal barrier function with a significant increase in TEWL by 1 hpi. The maximal mean TEWL (598.67 g/m^2^h) was recorded by 1 hpi and then declined, but at 24 hpi the mean value (396.75 g/m^2^h) was still significantly higher than the baseline value (20.54 g/m^2^h). The gradual decline of TEWL observed after 1 hpi may be caused by skin crust formation, which is an early characteristic of sheep scab pathology [3,40] thus limiting further transepidermal water loss. In man, TEWL is used as an indicator of impaired epidermal barrier function, however, reliability of TEWL as a marker for epidermal barrier function in other species, such as dogs, has been questioned [55]. TEWL does not appear to have been studied previously in sheep and the influence of ectoparasite infestation on TEWL has not been reported. Compromised barrier function in atopic dermatitis patients may be attributable to the proteolytic activity of *Der p 1* and *3*[15,16]. In sheep, the mouthparts of *P. ovis* may also impair barrier function by abrading the stratum corneum [56]. However, it is probable that *P. ovis* homologues of the HDM proteases *Der p 1* and *3*, such as *Pso o 1* and *3*, also contribute to the increase in TEWL by disruption of tight junctions. These and other proteases may further compromise barrier function by digestion of proteins such as filaggrin, loricrin and involucrin.

There is increasing evidence to suggest that defective skin barrier function in atopic dermatitis leads to a heightened immune response and increases the risk of allergic sensitisation [55,57,58]. The present study provides evidence of significant impairment of skin barrier function during a *P. ovis* infestation. As in the pathogenesis of atopic dermatitis it is probable that disruption of the epidermal barrier plays a critical role in the subsequent development of the immediate, late phase and delayed hypersensitivity reactions that characterise *P. ovis* infestation by facilitating increased transepidermal passage of mite products that provoke a Th2 dominated immune response [9].

In conclusion the present study has shown that within the first 24 h of infestation, *P. ovis* elicits a rapid and profound inflammatory response that is dominated by a polymorphonuclear infiltrate and severe epidermal pathology. This response is accompanied by significant decreases in the expression of a number of EDC genes, namely *FLG*, *IVL* and *LOR*, reduced presentation of filaggrin and loricrin protein at the epidermal surface and by a significant increase in TEWL. Many of these features are also documented in human and canine atopic dermatitis and suggest that sheep scab may provide a potential model for the study of certain aspects of the initial sensitisation phases during the pathogenesis of atopic dermatitis.

## Abbreviations

HDM: Huse dust mite; TEWL: Transepidermal water loss; NMF: Natural moisturising factor; NGS: Normal goat serum; EDC: Epidermal differentiation complex; MRI: Moredun Research Institute; Hpi: Hours post-infestation; MFI/mm: Mean fluorescence intensity/mm.

## Competing interests

The authors declare that they have no competing interests.

## Authors’ contributions

MS performed immunohistochemical and histological analysis and prepared the manuscript. TNM supervised the immunohistochemical analysis and performed statistical analysis, participated in the study design and coordination and contributed to the preparation of the manuscript. DF supervised immunohistochemical and qPCR analysis and contributed to the preparation of the manuscript. EJM designed and performed the qPCR analysis. AJN was involved in the cloning of the EDC gene fragments which were used in the qPCR analysis. AvdB participated in study design and coordination and contributed to the preparation of the manuscript. STGB performed the in vivo trial, collected skin biopsy samples, extracted RNA, performed the TEWL measurements and microarray data analysis, supervised the qPCR analysis, participated in study design and coordination and contributed to the preparation of the manuscript. All authors read and approved the final manuscript.
